# Bis(3,5-dimeth­oxy-2-{[2-(pyridin-2-yl)ethyl­imino-κ*N*]­meth­yl}phenolato-κ*O*)bis­(dimethyl sulfoxide)­manganese(III) perchlorate methanol 0.774-solvate

**DOI:** 10.1107/S205698901701204X

**Published:** 2017-09-15

**Authors:** Rita Egekenze, Yilma Gultneh, Ray J. Butcher

**Affiliations:** a Department of Chemistry, Howard University, 525 College Street NW, Washington, DC 20059, USA

**Keywords:** crystal structure, Mn^III^ Schiff base complex, Jahn–Teller distortion, DMSO coordination

## Abstract

The title compound, [Mn(C_16_H_17_N_2_O_3_)_2_(C_2_H_6_OS)_2_]ClO_4_·0.774CH_3_OH, comprises a central octa­hedrally coordinated Mn^III^ cation, with two bidentate Schiff base ligands occupying the equatorial positions and two dimethyl sulfoxide (DMSO) ligands occupying the axial positions. There are two independant cations in the asymmetric unit, with the Mn^III^ atoms of both cations being positioned on crystallographic centers of inversion. The perchlorate anion is disordered over two equivalent conformations, with occupancies of 0.744 (3) and 0.226 (3). In addition, there is a methanol solvent mol­ecule in the crystal lattice that is too close to the minor component of the perchlorate anion to be present simultaneously and thus it was refined to have the same occupancy as the major component of this anion. There is a Jahn–Teller distortion which results in Mn—O_DMSO_ axial bond lengths of 2.2365 (12) and 2.2368 (12) Å in the two cations. In the crystal, inter­molecular π–π stacking between the non-coordinating pyridine rings of each cation is observed. This π–π stacking, along with extensive O—H⋯O hydrogen bonding and C—H⋯O inter­actions, link the components into a complex three-dimensional array.

## Abstract 2

The title complex contains a central octa­hedrally coordinated Mn^III^ cation with to two bidentate Schiff base ligands occupying the equatorial positions and two dimethyl sulfoxide ligands occupying the axial positions. In addition, disordered perchlorate anions and solvent mol­ecules with a site-occupancy factor corresponding to that of the major fraction of the anions.

## Chemical context   

Single-mol­ecule magnets (SMMs) are a class of coordination compounds that attract a great deal of scientific attention because they exhibit magnetic bis­tability at low temperatures (Christou *et al.*, 2000 ▸; Gatteschi *et al.*, 2006 ▸). These finite size (zero-dimensional) mol­ecules possess a high-spin ground state *S_t_* and a magnetic anisotropy of the easy-axis type (negative zero-field splitting parameter *D*), which causes a slow relaxation of the magnetization at low temperatures, resulting in a hysteresis of the magnetization of purely mol­ecular origin (Sessoli *et al.*, 1993*a* ▸,*b* ▸; Gatteschi *et al.*, 1994 ▸; Aubin *et al.*, 1998 ▸; Gatteschi & Sessoli, 2003 ▸; Long, 2003 ▸; Thomas *et al.*, 1996 ▸). SMMs promise access to dynamic random access memory devices for quantum computing and to ultimate high-density memory storage devices in which each bit of digital information is stored on a single mol­ecule (Tejada, 2001 ▸; Awschalom *et al.*, 1992 ▸; Leuenberger & Loss, 2001 ▸; Cornia *et al.*, 2003 ▸; Dahlberg & Zhu, 1995 ▸).

The archetype of SMMs is the family of dodeca­nuclear manganese complexes, [Mn_12_O_12_(O_2_C*R*)_16_(OH_2_)_4_], Mn12 (Lis, 1980 ▸; Sessoli *et al.*, 1993*a* ▸,*b* ▸; Boyd *et al.*, 1988 ▸; Tsai *et al.*, 1994 ▸; Sun *et al.*, 1998 ▸; Boskovic *et al.*, 2002 ▸). Since the discovery of the SMM behavior of Mn12, a lot of synthetic effort has been devoted to the preparation of new mol­ecules with an increased anisotropy barrier. In this respect, it is inter­esting to note that already a dimeric Mn^III^ salen complex behaves as an SMM (Miyasaka *et al.*, 2004 ▸).

An undeveloped field in this chemistry is the use of manganese complexes of Schiff base ligands as precursors in the synthesis of SMMs. In a continuation of our studies in manganese chemistry with Schiff base ligands as precursors to SSMs (Egekenze *et al.*, 2017*a* ▸,*b* ▸,*c* ▸), we report the structure of bis­(3,5-dimeth­oxy-2-{[2-(pyridin-2-yl)ethyl­imino-κ*N*]meth­yl}phenolato-κ*O*)bis­(dimethyl sulfoxide)­manganese(III) per­chlorate methanol 0.774-solvate.
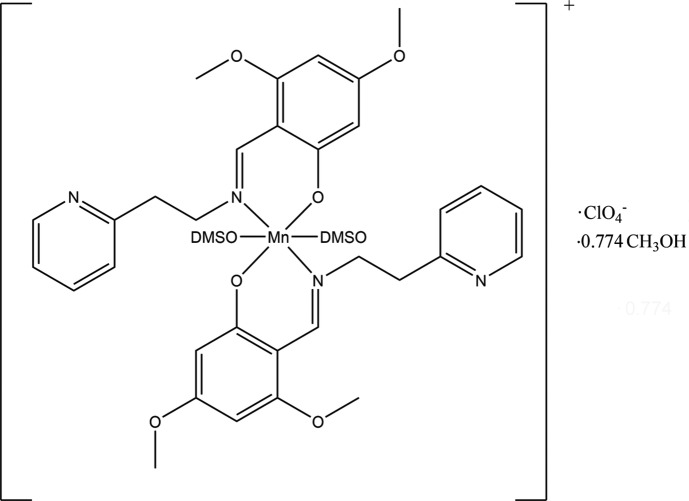


## Structural commentary   

**Figure 1:**
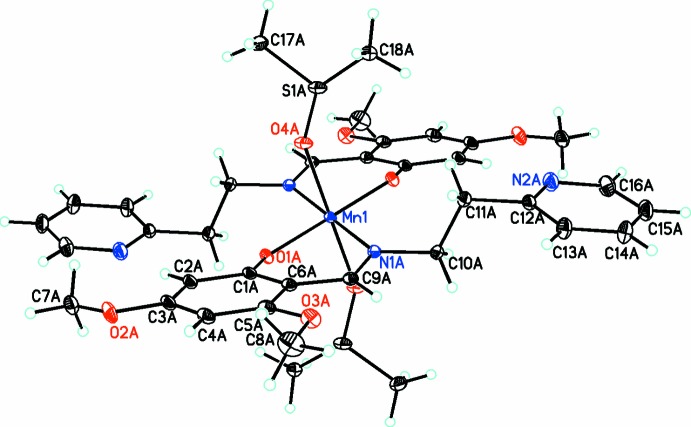
Diagram of one of the two equivalent cations, showing the atom labeling. Anions and solvent mol­ecules have been omitted for clarity. Atomic displacement parameters are at the 30% probability level.

In the structure of the title compound (Fig. 1 ▸), the cation contains a central octa­hedrally coordinated Mn^III^ cation, with two bidentate Schiff base ligands occupying the equatorial positions and two dimethyl sulfoxide (DMSO) ligands occupying the axial positions. There are two independant cations in the asymmetric unit, with the Mn^III^ atoms of both cations being positioned on crystallographic centers of inversion. The perchlorate anion is disordered over two equivalent conformations, with occupancies of 0.744 (3) and 0.226 (3). In addition, there is a disordered methanol solvent molecule in the crystal lattice.

Inter­estingly, the Schiff base ligand is potentially tridentate as it is the result of the condensation of 3,5-di­meth­oxy­salicyl­aldehyde with 2-(2-amino­eth­yl)pyridine. However, the pyridine arm does not coordinate to manganese and the softer N_py_-donors have been replaced by the O-donors of the DMSO mol­ecules as the complex was crystallized from DMSO. The Mn—O_phen_ [1.8757 (11) and 1.8770 (11) Å] and Mn—N_imine_ bond lengths [2.0335 (13) and 2.0380 (13) Å] are in the normal ranges found for manganese Schiff base complexes. As this is a high-spin *d* ^4^ cation, there is Jahn–Teller distortion (Jahn & Teller, 1937 ▸) which results in Mn—O_DMSO_ axial bond lengths of 2.2365 (12) and 2.2368 (12) Å in the two cations.

A survey of the Cambridge Structural Database (Groom *et al.*, 2016 ▸) for compounds of manganese Schiff base complexes with attached DMSO ligands showed only one other example (Glaser *et al.*, 2007 ▸) of a bis-DMSO complex of an Mn^III^ Schiff base. In this case, the DMSO ligands were also occupying axial positions. If the search was restricted to a single coordinating DMSO ligand, there was one relevant example (Bermejo *et al.*, 1994 ▸), aqua­[*N*,*N*′-bis­(3-bromo-5-nitro­salicyl­idene)-1,2-di­amino-(2-meth­yl)ethane](dimethyl sulfoxide)­manganese(II), which, however, contains both Mn^II^ and a tetra­dentate ligand and therefore no Jahn–Teller distorsion was observed.

## Supra­molecular features   

**Figure 2:**
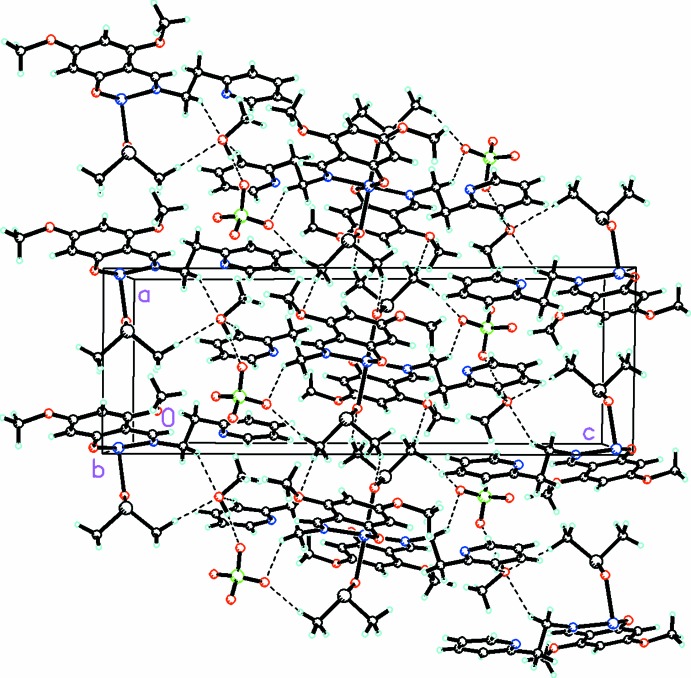
Packing diagram, viewed along the *b* axis, showing the extensive O—H⋯O and C—H⋯O inter­actions linking the cations, anions and solvent mol­ecules into a three-dimensional array. For the disordered moieties, only the major conformation is shown.

In the crystal structure, inter­molecular π–π stacking between the non-coordinating pyridine rings of each cation is observed with a perpendicular stacking distance of 3.623 Å and a slippage of 1.321 Å (symmetry code 1 − *x,*, 1 − *y*, 1 − *z*). This π–π stacking, along with extensive O—H⋯O hydrogen bonding and C—H⋯O inter­actions (Fig. 2 ▸ and Table 1 ▸), link the components into a complex three-dimensional array.

## Database survey   

A survey of the Cambridge Structural Database for examples of DMSO ligands coordinating to manganese Schiff base skeletons gave only one example of a bis-DMSO complex (refcode JETYOX) and only five examples with only one attached DMSO ligand (refcodes EBILOQ, FOFWIH, LEJCEI, WADZUZ, and WAFBAJ)

## Synthesis and crystallization   

### Synthesis of 3,5-dimeth­oxy-2-{[2-(pyridin-2-yl)ethyl­imino]­meth­yl}phenol   

A solution of 1.3985 g (11.4 mmol) of 2-(pyridin-2-yl)ethanamine in 15 ml of methanol was mixed with a solution of 2.0874 g (11.5 mmol) of 4,6-di­meth­oxy­salicylic aldehyde in 15 ml of methanol to obtain a dark-green solution. The solution was refluxed for 4 h. The thick dark-brown oil obtained was recrystallized from di­chloro­methane by slow evaporation of the solvent (yield: 3.02 g, 87%). Characterization data for C_16_H_18_N_2_O_3_ are as follows; mol­ecular mass: calculated for [C_16_H_19_N_2_O_3_]^+^ = 287.1396, ESI–MS determined *m*/*z* = 287.1390. IR (LiTaO_3_, KBr) (cm^−1^); 3008 (*w*), 2932 (*w*), 2850 (*w*), 1630 (*s*), 1610 (*s*), 1586 (*m*), 1564 (*m*), 1537 (*s*), 1470 (*m*), 1446 (*m-s*), 1434 (*s*), 1403 (*w*), 1354 (*s*), 1314 (*w*), 1290 (*w*), 1265 (*w*), 1231 (*m*), 1217 (*s*), 1201 (*s*), 1178 (*m*), 1144 (*s*), 1111 (*m*), 1100 (*m*), 1040 (*m*), 1010 (*m*), 986 (*m*), 929 (*m*), 879 (*m*), 824 (*s*), 771 (*s*), 746 (*m*), 690 (*w*), 655 (*m*). UV–Vis {λ_max_ (nm), (MeOH)}: 203 (180.31), 255 (23.19), 262 (25.43), 314 (137.83), 375 (35.61). ^1^H NMR {CDCl_3_}: δ 14.25 (*s*, 1H ArO-H), 8.58, (*s*, 1H, –CH=N), 5.50, 5.85, 7.25, 7.58, 8.28, (*s*, 1H ArH); 7.10 (*d*, 2H); 3.08 (*d*, 2H, CH_2_); 3.88 (*d*, 2H, CH_2_); 3.70 (*m*, 6H, 2(OCH_3_).

### Synthesis of bis­(3,5-dimeth­oxy-2-{[2-(pyridin-2-yl)ethyl­imino-κ*N*]­meth­yl}phenolato-κ*O*)bis­(dimethyl sulfoxide)­man­gan­ese(III) perchlorate methanol 0.774-solvate   

A solution of Mn(ClO_4_)_2_·6H_2_O (1.6965 g, 9.2 mmol) in methanol was added to a mixture of 3,5-dimeth­oxy-2-{[2-(pyridin-2-yl)ethyl­imino]­meth­yl}phenol (2.6252 g, 9.2 mmol) and tri­ethyl­amine (C_6_H_15_N; 1.65 ml, 9.2 mmol). The solution turned dark brown. It was refluxed for 4 h and cooled to room temperature. The solvent was reduced with a rotary evaporator and the resulting precipitate was filtered off by suction, washed with diethyl ether and dried in the desiccator. The precipitate was recrystallized from methanol and diethyl ether and crystals suitable for X-ray analysis were grown by slow evaporation of a DMSO solution in a yield of 2.89 g (67%). Characterization data for C_32_H_34_MnClN_4_O_10_ are: mol­ecular mass: calculated for [C_32_H_34_MnN_4_O_6_]^+^ = 625.1859, ESI–MS determined *m*/*z* = 625.2094. IR (LiTaO_3_, KBr) (cm^−1^): 294 (*w*), 1588 (*s*), 1543 (*m*), 1466 (*m*), 1450 (*m*), 1438 (*m*), 1417 (*m*), 1392 (*w*), 1338 (*m*), 1246 (*s*), 1220 (*s*), 1186 (*w*), 1165 (*s*), 1123 (*m*), 1081 (*s*), 1012 (*m*), 977 (*w*), 948 (*w*), 960 (*w*), 865 (*w*), 830 (*s*), 782 (*m*), 769 (*m*), 667 (*s*). UV–Vis [λ_max_ (nm), (MeOH)]: 204 (20074.07), 263 (9385.37), 307 (11149.44).

## Refinement   

Crystal data, data collection and structure refinement details are summarized in Table 2 ▸. The perchlorate anion is disordered over two equivalent conformations, with occupancies of 0.744 (3) and 0.226 (3). Both anions were constrained to have similar metrical and displacement parameters using both DFIX and SIMU commands in *SHELXL2016* (Sheldrick, 2015 ▸). In addition, there is a methanol solvent mol­ecule present. This mol­ecule is too close to the minor component of the perchlorate anion to be present simultaneously and thus it was refined to have the same occupancy as the major component of this anion. This model lowered the *R* factor by 0.4%. H atoms were positioned geometrically and allowed to ride on their parent atoms, with C—H distances ranging from 0.95 to 0.98 Å. *U*_iso_(H) = *xU*_eq_(C), where *x* = 1.5 for methyl H atoms and 1.2 for all other C-bound H atoms. The O—H hydrogen was refined isotropically.

## Supplementary Material

Crystal structure: contains datablock(s) I. DOI: 10.1107/S205698901701204X/im2481sup1.cif

Structure factors: contains datablock(s) I. DOI: 10.1107/S205698901701204X/im2481Isup2.hkl

CCDC reference: 1570012

Additional supporting information:  crystallographic information; 3D view; checkCIF report

## Figures and Tables

**Table 1:** Hydrogen-bond geometry (Å, °)

*D*—H⋯*A*	*D*—H	H⋯*A*	*D*⋯*A*	*D*—H⋯*A*
O1*S*—H1*S*⋯O14	0.83 (2)	2.21 (4)	2.864 (4)	136 (5)
O1*S*—H1*S*⋯O13*A*	0.83 (2)	1.06 (3)	1.813 (7)	147 (6)
C7*A*—H7*AA*⋯O12^i^	0.98	2.44	3.395 (3)	165
C7*A*—H7*AA*⋯O14*A* ^i^	0.98	2.49	3.415 (7)	157
C10*A*—H10*B*⋯O12^ii^	0.99	2.57	3.408 (3)	142
C10*A*—H10*B*⋯O14*A* ^ii^	0.99	2.36	3.243 (8)	148
C17*A*—H17*B*⋯O12	0.98	2.69	3.173 (4)	112
C9*B*—H9*BA*⋯O11^iii^	0.95	2.60	3.405 (3)	143
C9*B*—H9*BA*⋯O11*A* ^iii^	0.95	2.52	3.311 (7)	141
C16*A*—H16*A*⋯O12*A* ^iv^	0.95	2.66	3.563 (6)	160
C11*B*—H11*D*⋯O11*A* ^iii^	0.99	2.57	3.380 (8)	139
C17*A*—H17*A*⋯O3*A* ^v^	0.98	2.55	3.455 (3)	154
C13*A*—H13*A*⋯O1*S* ^ii^	0.95	2.42	3.343 (4)	165
C18*A*—H18*B*⋯O4*A* ^v^	0.98	2.56	3.491 (3)	160
C17*B*—H17*F*⋯O4*B* ^vi^	0.98	2.43	3.384 (2)	163
C7*B*—H7*BA*⋯O1*S* ^vii^	0.98	2.51	3.445 (4)	160
C10*B*—H10*D*⋯O1*S*	0.99	2.57	3.428 (3)	146
C18*B*—H18*F*⋯O1*S*	0.98	2.62	3.402 (3)	137

Symmetry codes: (i) 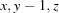; (ii) 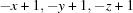; (iii) 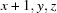; (iv) 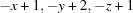; (v) 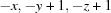; (vi) 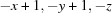; (vii) 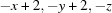.

**Table 2:** Experimental details

Crystal data	
Chemical formula	[Mn(C_16_H_17_N_2_O_3_)_2_(C_2_H_6_OS)_2_]ClO_4_·0.774CH_4_O
*M*_r_	906.10
Crystal system, space group	Triclinic, *P* 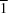
Temperature (K)	123
*a*, *b*, *c* (Å)	8.0730 (4), 11.0143 (4), 23.0453 (7)
α, β, γ (°)	87.540 (3), 89.175 (3), 87.729 (3)
*V* (Å^3^)	2045.49 (14)
*Z*	2
Radiation type	Mo *K*α
μ (mm^−1^)	0.56
Crystal size (mm)	0.71 × 0.40 × 0.24

Data collection	
Diffractometer	Agilent Xcalibur Ruby Gemini
Absorption correction	Analytical [*CrysAlis PRO* (Agilent, 2012 ▸), based on expressions derived by Clark & Reid (1995 ▸)]
*T*_min_, *T*_max_	0.754, 0.885
No. of measured, independent and observed [*I* > 2σ(*I*)] reflections	36920, 20464, 16259
*R*_int_	0.031
(sin θ/λ)_max_ (Å^−1^)	0.860

Refinement	
*R*[*F* ^2^ > 2σ(*F* ^2^)], *wR*(*F* ^2^), *S*	0.060, 0.149, 1.10
No. of reflections	20464
No. of parameters	577
No. of restraints	148
H-atom treatment	H atoms treated by a mixture of independent and constrained refinement
Δρ_max_, Δρ_min_ (e Å^−3^)	1.16, −1.01

Computer programs: *CrysAlis PRO* (Agilent, 2012 ▸), *SHELXS97* and *SHELXTL* (Sheldrick, 2008 ▸) and *SHELXL2016* (Sheldrick, 2015 ▸).

## supplementary crystallographic information

### Crystal data

**Table:**

[Mn(C_16_H_17_N_2_O_3_)_2_(C_2_H_6_OS)_2_]ClO_4_·0.774CH_4_O	*Z* = 2
*M**_r_* = 906.10	*F*(000) = 947.9
Triclinic, *P*1	*D*_x_ = 1.471 Mg m^−^^3^
*a* = 8.0730 (4) Å	Mo *K*α radiation, λ = 0.71073 Å
*b* = 11.0143 (4) Å	Cell parameters from 12258 reflections
*c* = 23.0453 (7) Å	θ = 3.1–37.6°
α = 87.540 (3)°	µ = 0.56 mm^−^^1^
β = 89.175 (3)°	*T* = 123 K
γ = 87.729 (3)°	Prism, red-brown
*V* = 2045.49 (14) Å^3^	0.71 × 0.40 × 0.24 mm

### Data collection

**Table:**

Agilent Xcalibur Ruby Gemini diffractometer	16259 reflections with *I* > 2σ(*I*)
Detector resolution: 10.5081 pixels mm^-1^	*R*_int_ = 0.031
ω scans	θ_max_ = 37.7°, θ_min_ = 3.1°
Absorption correction: analytical [CrysAlis PRO (Agilent, 2012), based on expressions derived by Clark & Reid (1995)]	*h* = −13→13
*T*_min_ = 0.754, *T*_max_ = 0.885	*k* = −18→18
36920 measured reflections	*l* = −39→37
20464 independent reflections	

### Refinement

**Table:**

Refinement on *F*^2^	Primary atom site location: structure-invariant direct methods
Least-squares matrix: full	Secondary atom site location: difference Fourier map
*R*[*F*^2^ > 2σ(*F*^2^)] = 0.060	Hydrogen site location: mixed
*wR*(*F*^2^) = 0.149	H atoms treated by a mixture of independent and constrained refinement
*S* = 1.10	*w* = 1/[σ^2^(*F*_o_^2^) + (0.0465*P*)^2^ + 1.6285*P*] where *P* = (*F*_o_^2^ + 2*F*_c_^2^)/3
20464 reflections	(Δ/σ)_max_ = 0.001
577 parameters	Δρ_max_ = 1.16 e Å^−^^3^
148 restraints	Δρ_min_ = −1.01 e Å^−^^3^

### Special details

**Table:**

Geometry. All esds (except the esd in the dihedral angle between two l.s. planes) are estimated using the full covariance matrix. The cell esds are taken into account individually in the estimation of esds in distances, angles and torsion angles; correlations between esds in cell parameters are only used when they are defined by crystal symmetry. An approximate (isotropic) treatment of cell esds is used for estimating esds involving l.s. planes.

### Fractional atomic coordinates and isotropic or equivalent isotropic displacement parameters (Å^2^)

**Table:**

	*x*	*y*	*z*	*U*_iso_*/*U*_eq_	Occ. (<1)
Mn1	0.500000	0.500000	0.500000	0.01344 (6)	
Mn2	1.000000	0.500000	0.000000	0.01420 (6)	
S1A	0.17543 (5)	0.65456 (4)	0.46048 (2)	0.02256 (8)	
S1B	0.67033 (5)	0.66578 (4)	0.02599 (2)	0.02310 (8)	
Cl1	0.30171 (12)	0.78497 (7)	0.25281 (4)	0.03133 (18)	0.774 (3)
O11	0.3026 (4)	0.7096 (2)	0.20426 (11)	0.0591 (14)	0.774 (3)
O12	0.2546 (4)	0.7170 (2)	0.30416 (11)	0.0419 (8)	0.774 (3)
O13	0.1894 (3)	0.88677 (19)	0.24370 (10)	0.0649 (10)	0.774 (3)
O14	0.4653 (3)	0.8281 (3)	0.26037 (13)	0.0809 (11)	0.774 (3)
Cl1A	0.3726 (7)	0.7628 (3)	0.24715 (13)	0.0490 (10)	0.226 (3)
O11A	0.3059 (10)	0.7049 (7)	0.1990 (3)	0.044 (3)	0.226 (3)
O12A	0.3376 (13)	0.8908 (3)	0.2415 (3)	0.064 (3)	0.226 (3)
O13A	0.5479 (6)	0.7399 (9)	0.2486 (4)	0.096 (4)	0.226 (3)
O14A	0.2998 (11)	0.7157 (7)	0.2999 (2)	0.0313 (19)	0.226 (3)
O1A	0.50038 (15)	0.34259 (10)	0.47199 (5)	0.0179 (2)	
O2A	0.2589 (2)	−0.03544 (13)	0.43949 (7)	0.0313 (3)	
O3A	0.24473 (18)	0.12275 (12)	0.62751 (6)	0.0267 (3)	
O4A	0.22922 (15)	0.52580 (11)	0.48181 (6)	0.0217 (2)	
O1B	1.01133 (16)	0.65127 (10)	−0.04028 (5)	0.0190 (2)	
O2B	1.2190 (2)	1.03701 (12)	−0.09960 (6)	0.0279 (3)	
O3B	1.22953 (19)	0.90515 (12)	0.09819 (6)	0.0255 (3)	
O4B	0.72827 (15)	0.53481 (11)	0.01510 (6)	0.0232 (2)	
N1A	0.45577 (16)	0.43169 (12)	0.58189 (6)	0.0157 (2)	
N2A	0.4453 (2)	0.76067 (16)	0.69461 (8)	0.0294 (3)	
N1B	1.04014 (16)	0.58273 (12)	0.07568 (6)	0.0159 (2)	
N2B	1.0744 (3)	0.25942 (16)	0.20333 (7)	0.0311 (4)	
C1A	0.41715 (19)	0.24823 (13)	0.49218 (7)	0.0158 (2)	
C2A	0.3824 (2)	0.15812 (14)	0.45271 (7)	0.0191 (3)	
H2AA	0.414312	0.167122	0.412964	0.023*	
C3A	0.3007 (2)	0.05629 (14)	0.47333 (8)	0.0218 (3)	
C4A	0.2513 (2)	0.04030 (15)	0.53187 (8)	0.0229 (3)	
H4AA	0.194402	−0.029901	0.544822	0.027*	
C5A	0.2869 (2)	0.12795 (14)	0.57000 (7)	0.0193 (3)	
C6A	0.36881 (19)	0.23522 (13)	0.55126 (7)	0.0162 (2)	
C7A	0.3153 (3)	−0.03191 (19)	0.38039 (10)	0.0318 (4)	
H7AA	0.276708	−0.102948	0.361093	0.048*	
H7AB	0.271203	0.042507	0.360392	0.048*	
H7AC	0.436765	−0.032877	0.379174	0.048*	
C8A	0.1632 (3)	0.0166 (2)	0.64944 (11)	0.0411 (5)	
H8AA	0.139570	0.023690	0.691014	0.062*	
H8AB	0.059117	0.009237	0.628745	0.062*	
H8AC	0.235214	−0.055674	0.643549	0.062*	
C9A	0.40744 (19)	0.32197 (14)	0.59325 (7)	0.0167 (2)	
H9AA	0.396694	0.296874	0.633040	0.020*	
C10A	0.4913 (2)	0.50364 (15)	0.63280 (7)	0.0185 (3)	
H10A	0.584878	0.556669	0.623176	0.022*	
H10B	0.525077	0.447738	0.665681	0.022*	
C11A	0.3406 (2)	0.58216 (17)	0.65116 (8)	0.0236 (3)	
H11A	0.308632	0.640228	0.618897	0.028*	
H11B	0.245813	0.529760	0.659978	0.028*	
C12A	0.3801 (2)	0.65134 (16)	0.70419 (8)	0.0218 (3)	
C13A	0.3539 (3)	0.60108 (19)	0.75975 (8)	0.0296 (4)	
H13A	0.307551	0.523374	0.765017	0.036*	
C14A	0.3962 (3)	0.6655 (2)	0.80748 (9)	0.0334 (4)	
H14A	0.378686	0.632966	0.845823	0.040*	
C15A	0.4643 (3)	0.7779 (2)	0.79808 (9)	0.0332 (4)	
H15A	0.495327	0.824044	0.829790	0.040*	
C16A	0.4863 (3)	0.8215 (2)	0.74155 (10)	0.0350 (4)	
H16A	0.533045	0.898859	0.735357	0.042*	
C17A	0.0194 (3)	0.6319 (2)	0.40838 (9)	0.0303 (4)	
H17A	−0.022353	0.710897	0.392337	0.045*	
H17B	0.066872	0.583731	0.376985	0.045*	
H17C	−0.071933	0.588592	0.427203	0.045*	
C18A	0.0442 (3)	0.71209 (18)	0.51712 (9)	0.0286 (4)	
H18A	0.004811	0.795149	0.506235	0.043*	
H18B	−0.050952	0.660124	0.522905	0.043*	
H18C	0.106810	0.712481	0.553218	0.043*	
C1B	1.08105 (19)	0.75281 (13)	−0.02710 (7)	0.0160 (2)	
C2B	1.1110 (2)	0.83936 (14)	−0.07256 (7)	0.0182 (3)	
H2BA	1.082866	0.823997	−0.111375	0.022*	
C3B	1.1824 (2)	0.94740 (14)	−0.05957 (7)	0.0196 (3)	
C4B	1.2262 (2)	0.97316 (14)	−0.00270 (7)	0.0204 (3)	
H4BA	1.276857	1.047111	0.004956	0.025*	
C5B	1.1944 (2)	0.88913 (14)	0.04182 (7)	0.0182 (3)	
C6B	1.12204 (19)	0.77650 (13)	0.03091 (7)	0.0161 (2)	
C7B	1.1637 (3)	1.02612 (19)	−0.15781 (9)	0.0314 (4)	
H7BA	1.198245	1.096404	−0.181871	0.047*	
H7BB	1.212344	0.951376	−0.173589	0.047*	
H7BC	1.042495	1.023133	−0.157835	0.047*	
C8B	1.3098 (3)	1.01347 (18)	0.11256 (9)	0.0296 (4)	
H8BA	1.327350	1.012738	0.154572	0.044*	
H8BB	1.417031	1.016876	0.092174	0.044*	
H8BC	1.240046	1.084849	0.100772	0.044*	
C9B	1.08844 (19)	0.69338 (14)	0.07878 (7)	0.0169 (2)	
H9BA	1.102943	0.722059	0.116600	0.020*	
C10B	1.0104 (2)	0.51639 (14)	0.13192 (7)	0.0182 (3)	
H10C	0.917631	0.461323	0.127936	0.022*	
H10D	0.978321	0.575305	0.161728	0.022*	
C11B	1.1657 (2)	0.44193 (16)	0.15167 (7)	0.0212 (3)	
H11C	1.200209	0.384829	0.121331	0.025*	
H11D	1.257509	0.497173	0.157111	0.025*	
C12B	1.1317 (2)	0.37149 (15)	0.20787 (7)	0.0200 (3)	
C13B	1.1580 (2)	0.42174 (17)	0.26116 (8)	0.0248 (3)	
H13B	1.200848	0.500606	0.262816	0.030*	
C14B	1.1211 (3)	0.35536 (18)	0.31177 (8)	0.0260 (3)	
H14B	1.137798	0.388033	0.348626	0.031*	
C15B	1.0593 (3)	0.24032 (19)	0.30771 (8)	0.0282 (4)	
H15B	1.030838	0.193012	0.341571	0.034*	
C16B	1.0403 (3)	0.1966 (2)	0.25299 (9)	0.0371 (5)	
H16B	1.000665	0.116938	0.250348	0.045*	
C17B	0.5226 (2)	0.70393 (18)	−0.02905 (9)	0.0264 (3)	
H17D	0.479662	0.787575	−0.024736	0.040*	
H17E	0.575775	0.697021	−0.067328	0.040*	
H17F	0.430998	0.648204	−0.025410	0.040*	
C18B	0.5303 (3)	0.6533 (3)	0.08640 (9)	0.0400 (5)	
H18D	0.487442	0.734523	0.096103	0.060*	
H18E	0.437993	0.603326	0.076394	0.060*	
H18F	0.588691	0.615228	0.119890	0.060*	
O1S	0.7366 (3)	0.6892 (3)	0.21182 (12)	0.0490 (6)	0.774 (3)
H1S	0.644 (2)	0.689 (5)	0.2281 (13)	0.073*	0.774 (3)
C1S	0.8497 (6)	0.7039 (4)	0.25684 (15)	0.0523 (9)	0.774 (3)
H1SD	0.958649	0.721944	0.240058	0.078*	0.774 (3)
H1SA	0.858738	0.628816	0.281184	0.078*	0.774 (3)
H1SB	0.810224	0.771155	0.280573	0.078*	0.774 (3)

### Atomic displacement parameters (Å^2^)

**Table:**

	*U*^11^	*U*^22^	*U*^33^	*U*^12^	*U*^13^	*U*^23^
Mn1	0.01323 (13)	0.00974 (12)	0.01742 (13)	−0.00149 (10)	−0.00032 (9)	−0.00011 (9)
Mn2	0.01386 (13)	0.01008 (12)	0.01888 (14)	−0.00177 (10)	−0.00235 (10)	−0.00110 (10)
S1A	0.01467 (16)	0.01704 (17)	0.0354 (2)	0.00072 (13)	−0.00139 (14)	0.00494 (14)
S1B	0.01565 (17)	0.01864 (17)	0.0354 (2)	0.00003 (13)	−0.00061 (14)	−0.00671 (15)
Cl1	0.0454 (5)	0.0229 (3)	0.0253 (3)	0.0047 (3)	−0.0010 (3)	−0.0018 (2)
O11	0.100 (3)	0.051 (3)	0.0289 (16)	−0.013 (2)	−0.0065 (19)	−0.0146 (16)
O12	0.069 (2)	0.0277 (13)	0.0291 (13)	−0.0005 (12)	0.0004 (12)	0.0017 (10)
O13	0.088 (2)	0.0512 (16)	0.0505 (15)	0.0363 (16)	0.0060 (15)	0.0154 (12)
O14	0.0537 (19)	0.084 (3)	0.110 (3)	−0.0289 (18)	0.0138 (18)	−0.045 (2)
Cl1A	0.096 (3)	0.0244 (13)	0.0278 (12)	−0.0124 (16)	−0.0050 (16)	−0.0059 (9)
O11A	0.065 (7)	0.038 (6)	0.028 (5)	0.009 (6)	0.018 (5)	−0.014 (4)
O12A	0.128 (8)	0.022 (3)	0.043 (4)	−0.009 (5)	−0.006 (5)	0.006 (3)
O13A	0.072 (8)	0.117 (10)	0.101 (8)	−0.004 (8)	0.007 (7)	−0.017 (8)
O14A	0.055 (5)	0.023 (4)	0.016 (3)	0.009 (3)	−0.013 (3)	0.000 (2)
O1A	0.0215 (5)	0.0115 (4)	0.0210 (5)	−0.0037 (4)	0.0028 (4)	−0.0012 (4)
O2A	0.0367 (8)	0.0171 (6)	0.0415 (8)	−0.0086 (5)	0.0009 (6)	−0.0113 (5)
O3A	0.0313 (7)	0.0201 (6)	0.0285 (6)	−0.0068 (5)	0.0060 (5)	0.0056 (5)
O4A	0.0146 (5)	0.0153 (5)	0.0348 (6)	0.0003 (4)	−0.0022 (4)	0.0034 (4)
O1B	0.0233 (6)	0.0124 (4)	0.0218 (5)	−0.0039 (4)	−0.0057 (4)	0.0001 (4)
O2B	0.0399 (8)	0.0182 (5)	0.0259 (6)	−0.0097 (5)	−0.0018 (5)	0.0050 (4)
O3B	0.0363 (7)	0.0190 (5)	0.0226 (6)	−0.0126 (5)	−0.0047 (5)	−0.0039 (4)
O4B	0.0149 (5)	0.0160 (5)	0.0390 (7)	−0.0006 (4)	−0.0005 (4)	−0.0043 (5)
N1A	0.0147 (5)	0.0143 (5)	0.0181 (5)	−0.0013 (4)	−0.0020 (4)	−0.0005 (4)
N2A	0.0367 (9)	0.0231 (7)	0.0291 (8)	−0.0030 (6)	−0.0023 (6)	−0.0052 (6)
N1B	0.0147 (5)	0.0133 (5)	0.0197 (5)	−0.0023 (4)	−0.0008 (4)	−0.0005 (4)
N2B	0.0472 (11)	0.0226 (7)	0.0241 (7)	−0.0117 (7)	−0.0045 (6)	0.0013 (5)
C1A	0.0143 (6)	0.0102 (5)	0.0228 (6)	−0.0004 (4)	−0.0011 (5)	−0.0001 (4)
C2A	0.0193 (7)	0.0128 (6)	0.0254 (7)	−0.0007 (5)	−0.0011 (5)	−0.0031 (5)
C3A	0.0202 (7)	0.0121 (6)	0.0335 (8)	−0.0015 (5)	−0.0017 (6)	−0.0047 (5)
C4A	0.0205 (7)	0.0117 (6)	0.0366 (9)	−0.0037 (5)	−0.0002 (6)	0.0003 (6)
C5A	0.0167 (6)	0.0142 (6)	0.0268 (7)	−0.0009 (5)	0.0012 (5)	0.0034 (5)
C6A	0.0147 (6)	0.0119 (5)	0.0218 (6)	−0.0015 (4)	−0.0002 (5)	0.0011 (4)
C7A	0.0351 (10)	0.0231 (8)	0.0384 (10)	−0.0021 (7)	−0.0053 (8)	−0.0120 (7)
C8A	0.0475 (14)	0.0286 (10)	0.0466 (13)	−0.0147 (9)	0.0098 (10)	0.0142 (9)
C9A	0.0152 (6)	0.0152 (6)	0.0194 (6)	−0.0009 (5)	−0.0007 (4)	0.0025 (5)
C10A	0.0186 (7)	0.0185 (6)	0.0188 (6)	−0.0015 (5)	−0.0040 (5)	−0.0027 (5)
C11A	0.0210 (7)	0.0270 (8)	0.0234 (7)	0.0021 (6)	−0.0044 (5)	−0.0082 (6)
C12A	0.0204 (7)	0.0215 (7)	0.0239 (7)	0.0012 (6)	−0.0031 (5)	−0.0076 (5)
C13A	0.0351 (10)	0.0295 (9)	0.0255 (8)	−0.0099 (8)	−0.0023 (7)	−0.0069 (7)
C14A	0.0397 (11)	0.0383 (11)	0.0235 (8)	−0.0085 (9)	−0.0038 (7)	−0.0074 (7)
C15A	0.0343 (10)	0.0344 (10)	0.0327 (10)	−0.0048 (8)	−0.0057 (8)	−0.0149 (8)
C16A	0.0420 (12)	0.0241 (9)	0.0400 (11)	−0.0069 (8)	−0.0043 (9)	−0.0095 (8)
C17A	0.0280 (9)	0.0348 (10)	0.0272 (9)	0.0094 (8)	−0.0050 (7)	−0.0014 (7)
C18A	0.0282 (9)	0.0265 (8)	0.0313 (9)	0.0070 (7)	−0.0079 (7)	−0.0063 (7)
C1B	0.0153 (6)	0.0112 (5)	0.0215 (6)	−0.0009 (4)	−0.0019 (5)	−0.0009 (4)
C2B	0.0207 (7)	0.0135 (6)	0.0204 (6)	−0.0016 (5)	−0.0019 (5)	0.0002 (5)
C3B	0.0214 (7)	0.0129 (6)	0.0246 (7)	−0.0025 (5)	−0.0009 (5)	0.0013 (5)
C4B	0.0229 (7)	0.0132 (6)	0.0256 (7)	−0.0050 (5)	−0.0016 (5)	−0.0015 (5)
C5B	0.0182 (6)	0.0151 (6)	0.0216 (7)	−0.0032 (5)	−0.0016 (5)	−0.0025 (5)
C6B	0.0164 (6)	0.0126 (5)	0.0195 (6)	−0.0024 (5)	−0.0013 (5)	−0.0017 (4)
C7B	0.0429 (12)	0.0257 (8)	0.0253 (8)	−0.0046 (8)	−0.0002 (7)	0.0045 (7)
C8B	0.0391 (11)	0.0224 (8)	0.0288 (9)	−0.0146 (7)	−0.0058 (7)	−0.0045 (6)
C9B	0.0166 (6)	0.0142 (6)	0.0201 (6)	−0.0017 (5)	−0.0018 (5)	−0.0015 (5)
C10B	0.0194 (7)	0.0157 (6)	0.0196 (6)	−0.0029 (5)	0.0013 (5)	0.0001 (5)
C11B	0.0192 (7)	0.0224 (7)	0.0218 (7)	−0.0021 (6)	−0.0001 (5)	0.0034 (5)
C12B	0.0198 (7)	0.0185 (6)	0.0216 (7)	−0.0019 (5)	−0.0020 (5)	0.0023 (5)
C13B	0.0302 (9)	0.0204 (7)	0.0243 (8)	−0.0069 (6)	−0.0030 (6)	0.0001 (6)
C14B	0.0317 (9)	0.0256 (8)	0.0209 (7)	−0.0048 (7)	−0.0024 (6)	0.0003 (6)
C15B	0.0344 (10)	0.0279 (9)	0.0223 (8)	−0.0080 (7)	−0.0018 (6)	0.0051 (6)
C16B	0.0601 (15)	0.0241 (9)	0.0284 (9)	−0.0192 (9)	−0.0044 (9)	0.0026 (7)
C17B	0.0242 (8)	0.0235 (8)	0.0310 (9)	0.0027 (6)	0.0021 (6)	0.0020 (6)
C18B	0.0375 (12)	0.0538 (14)	0.0267 (9)	0.0186 (10)	0.0031 (8)	0.0007 (9)
O1S	0.0477 (15)	0.0439 (14)	0.0552 (15)	0.0005 (11)	0.0035 (11)	−0.0039 (11)
C1S	0.077 (3)	0.0470 (19)	0.0332 (16)	0.0012 (19)	0.0020 (15)	−0.0047 (13)

### Geometric parameters (Å, º)

**Table:**

Mn1—O1A	1.8757 (11)	C10A—H10A	0.9900
Mn1—O1A^i^	1.8757 (11)	C10A—H10B	0.9900
Mn1—N1A	2.0335 (13)	C11A—C12A	1.512 (2)
Mn1—N1A^i^	2.0336 (13)	C11A—H11A	0.9900
Mn1—O4A	2.2365 (12)	C11A—H11B	0.9900
Mn1—O4A^i^	2.2365 (12)	C12A—C13A	1.390 (3)
Mn2—O1B	1.8770 (11)	C13A—C14A	1.388 (3)
Mn2—O1B^ii^	1.8770 (11)	C13A—H13A	0.9500
Mn2—N1B^ii^	2.0380 (13)	C14A—C15A	1.382 (3)
Mn2—N1B	2.0380 (13)	C14A—H14A	0.9500
Mn2—O4B^ii^	2.2368 (13)	C15A—C16A	1.381 (3)
Mn2—O4B	2.2368 (13)	C15A—H15A	0.9500
S1A—O4A	1.5293 (12)	C16A—H16A	0.9500
S1A—C17A	1.785 (2)	C17A—H17A	0.9800
S1A—C18A	1.791 (2)	C17A—H17B	0.9800
S1B—O4B	1.5287 (13)	C17A—H17C	0.9800
S1B—C17B	1.779 (2)	C18A—H18A	0.9800
S1B—C18B	1.785 (2)	C18A—H18B	0.9800
Cl1—O11	1.4212 (15)	C18A—H18C	0.9800
Cl1—O13	1.4241 (14)	C1B—C2B	1.411 (2)
Cl1—O12	1.4279 (15)	C1B—C6B	1.419 (2)
Cl1—O14	1.4365 (15)	C2B—C3B	1.388 (2)
Cl1A—O11A	1.4256 (15)	C2B—H2BA	0.9500
Cl1A—O12A	1.4274 (15)	C3B—C4B	1.406 (2)
Cl1A—O14A	1.4280 (15)	C4B—C5B	1.380 (2)
Cl1A—O13A	1.4285 (15)	C4B—H4BA	0.9500
O1A—C1A	1.3240 (18)	C5B—C6B	1.425 (2)
O2A—C3A	1.358 (2)	C6B—C9B	1.433 (2)
O2A—C7A	1.429 (3)	C7B—H7BA	0.9800
O3A—C5A	1.363 (2)	C7B—H7BB	0.9800
O3A—C8A	1.434 (2)	C7B—H7BC	0.9800
O1B—C1B	1.3206 (18)	C8B—H8BA	0.9800
O2B—C3B	1.360 (2)	C8B—H8BB	0.9800
O2B—C7B	1.431 (2)	C8B—H8BC	0.9800
O3B—C5B	1.354 (2)	C9B—H9BA	0.9500
O3B—C8B	1.434 (2)	C10B—C11B	1.534 (2)
N1A—C9A	1.299 (2)	C10B—H10C	0.9900
N1A—C10A	1.481 (2)	C10B—H10D	0.9900
N2A—C12A	1.342 (3)	C11B—C12B	1.508 (2)
N2A—C16A	1.349 (3)	C11B—H11C	0.9900
N1B—C9B	1.299 (2)	C11B—H11D	0.9900
N1B—C10B	1.481 (2)	C12B—C13B	1.391 (2)
N2B—C16B	1.343 (3)	C13B—C14B	1.385 (3)
N2B—C12B	1.344 (2)	C13B—H13B	0.9500
C1A—C2A	1.414 (2)	C14B—C15B	1.387 (3)
C1A—C6A	1.414 (2)	C14B—H14B	0.9500
C2A—C3A	1.387 (2)	C15B—C16B	1.382 (3)
C2A—H2AA	0.9500	C15B—H15B	0.9500
C3A—C4A	1.407 (3)	C16B—H16B	0.9500
C4A—C5A	1.374 (2)	C17B—H17D	0.9800
C4A—H4AA	0.9500	C17B—H17E	0.9800
C5A—C6A	1.425 (2)	C17B—H17F	0.9800
C6A—C9A	1.435 (2)	C18B—H18D	0.9800
C7A—H7AA	0.9800	C18B—H18E	0.9800
C7A—H7AB	0.9800	C18B—H18F	0.9800
C7A—H7AC	0.9800	O1S—C1S	1.410 (5)
C8A—H8AA	0.9800	O1S—H1S	0.830 (10)
C8A—H8AB	0.9800	C1S—H1SD	0.9800
C8A—H8AC	0.9800	C1S—H1SA	0.9800
C9A—H9AA	0.9500	C1S—H1SB	0.9800
C10A—C11A	1.530 (2)		
			
O1A—Mn1—O1A^i^	180.0	H11A—C11A—H11B	108.1
O1A—Mn1—N1A	90.10 (5)	N2A—C12A—C13A	122.45 (16)
O1A^i^—Mn1—N1A	89.90 (5)	N2A—C12A—C11A	116.68 (16)
O1A—Mn1—N1A^i^	89.90 (5)	C13A—C12A—C11A	120.84 (17)
O1A^i^—Mn1—N1A^i^	90.10 (5)	C14A—C13A—C12A	119.36 (19)
N1A—Mn1—N1A^i^	180.00 (8)	C14A—C13A—H13A	120.3
O1A—Mn1—O4A	90.48 (5)	C12A—C13A—H13A	120.3
O1A^i^—Mn1—O4A	89.52 (5)	C15A—C14A—C13A	118.7 (2)
N1A—Mn1—O4A	92.32 (5)	C15A—C14A—H14A	120.7
N1A^i^—Mn1—O4A	87.68 (5)	C13A—C14A—H14A	120.7
O1A—Mn1—O4A^i^	89.52 (5)	C16A—C15A—C14A	118.46 (18)
O1A^i^—Mn1—O4A^i^	90.48 (5)	C16A—C15A—H15A	120.8
N1A—Mn1—O4A^i^	87.68 (5)	C14A—C15A—H15A	120.8
N1A^i^—Mn1—O4A^i^	92.32 (5)	N2A—C16A—C15A	123.8 (2)
O4A—Mn1—O4A^i^	180.0	N2A—C16A—H16A	118.1
O1B—Mn2—O1B^ii^	180.00 (8)	C15A—C16A—H16A	118.1
O1B—Mn2—N1B^ii^	90.60 (5)	S1A—C17A—H17A	109.5
O1B^ii^—Mn2—N1B^ii^	89.40 (5)	S1A—C17A—H17B	109.5
O1B—Mn2—N1B	89.40 (5)	H17A—C17A—H17B	109.5
O1B^ii^—Mn2—N1B	90.59 (5)	S1A—C17A—H17C	109.5
N1B^ii^—Mn2—N1B	180.00 (7)	H17A—C17A—H17C	109.5
O1B—Mn2—O4B^ii^	90.15 (5)	H17B—C17A—H17C	109.5
O1B^ii^—Mn2—O4B^ii^	89.85 (5)	S1A—C18A—H18A	109.5
N1B^ii^—Mn2—O4B^ii^	87.99 (5)	S1A—C18A—H18B	109.5
N1B—Mn2—O4B^ii^	92.01 (5)	H18A—C18A—H18B	109.5
O1B—Mn2—O4B	89.85 (5)	S1A—C18A—H18C	109.5
O1B^ii^—Mn2—O4B	90.15 (5)	H18A—C18A—H18C	109.5
N1B^ii^—Mn2—O4B	92.01 (5)	H18B—C18A—H18C	109.5
N1B—Mn2—O4B	87.99 (5)	O1B—C1B—C2B	117.87 (14)
O4B^ii^—Mn2—O4B	180.0	O1B—C1B—C6B	121.68 (14)
O4A—S1A—C17A	104.11 (9)	C2B—C1B—C6B	120.43 (14)
O4A—S1A—C18A	105.08 (9)	C3B—C2B—C1B	118.84 (14)
C17A—S1A—C18A	98.35 (9)	C3B—C2B—H2BA	120.6
O4B—S1B—C17B	104.91 (9)	C1B—C2B—H2BA	120.6
O4B—S1B—C18B	104.63 (10)	O2B—C3B—C2B	124.31 (15)
C17B—S1B—C18B	98.31 (10)	O2B—C3B—C4B	113.50 (14)
O11—Cl1—O13	110.35 (8)	C2B—C3B—C4B	122.18 (15)
O11—Cl1—O12	109.97 (8)	C5B—C4B—C3B	118.84 (14)
O13—Cl1—O12	109.65 (8)	C5B—C4B—H4BA	120.6
O11—Cl1—O14	109.25 (8)	C3B—C4B—H4BA	120.6
O13—Cl1—O14	108.89 (8)	O3B—C5B—C4B	123.81 (14)
O12—Cl1—O14	108.71 (8)	O3B—C5B—C6B	114.89 (14)
O11A—Cl1A—O12A	109.64 (9)	C4B—C5B—C6B	121.30 (14)
O11A—Cl1A—O14A	109.60 (9)	C1B—C6B—C5B	118.39 (14)
O12A—Cl1A—O14A	109.38 (9)	C1B—C6B—C9B	122.46 (13)
O11A—Cl1A—O13A	109.48 (9)	C5B—C6B—C9B	119.13 (14)
O12A—Cl1A—O13A	109.41 (9)	O2B—C7B—H7BA	109.5
O14A—Cl1A—O13A	109.32 (9)	O2B—C7B—H7BB	109.5
C1A—O1A—Mn1	128.49 (10)	H7BA—C7B—H7BB	109.5
C3A—O2A—C7A	117.94 (16)	O2B—C7B—H7BC	109.5
C5A—O3A—C8A	117.26 (17)	H7BA—C7B—H7BC	109.5
S1A—O4A—Mn1	114.48 (7)	H7BB—C7B—H7BC	109.5
C1B—O1B—Mn2	131.56 (10)	O3B—C8B—H8BA	109.5
C3B—O2B—C7B	118.09 (15)	O3B—C8B—H8BB	109.5
C5B—O3B—C8B	118.46 (14)	H8BA—C8B—H8BB	109.5
S1B—O4B—Mn2	116.94 (7)	O3B—C8B—H8BC	109.5
C9A—N1A—C10A	116.07 (13)	H8BA—C8B—H8BC	109.5
C9A—N1A—Mn1	123.36 (11)	H8BB—C8B—H8BC	109.5
C10A—N1A—Mn1	120.39 (10)	N1B—C9B—C6B	126.56 (14)
C12A—N2A—C16A	117.25 (18)	N1B—C9B—H9BA	116.7
C9B—N1B—C10B	115.93 (13)	C6B—C9B—H9BA	116.7
C9B—N1B—Mn2	124.38 (11)	N1B—C10B—C11B	111.19 (13)
C10B—N1B—Mn2	119.67 (10)	N1B—C10B—H10C	109.4
C16B—N2B—C12B	117.14 (17)	C11B—C10B—H10C	109.4
O1A—C1A—C2A	117.43 (14)	N1B—C10B—H10D	109.4
O1A—C1A—C6A	121.76 (14)	C11B—C10B—H10D	109.4
C2A—C1A—C6A	120.78 (14)	H10C—C10B—H10D	108.0
C3A—C2A—C1A	118.47 (16)	C12B—C11B—C10B	110.56 (14)
C3A—C2A—H2AA	120.8	C12B—C11B—H11C	109.5
C1A—C2A—H2AA	120.8	C10B—C11B—H11C	109.5
O2A—C3A—C2A	123.82 (17)	C12B—C11B—H11D	109.5
O2A—C3A—C4A	113.97 (15)	C10B—C11B—H11D	109.5
C2A—C3A—C4A	122.21 (15)	H11C—C11B—H11D	108.1
C5A—C4A—C3A	118.87 (15)	N2B—C12B—C13B	122.58 (16)
C5A—C4A—H4AA	120.6	N2B—C12B—C11B	116.46 (15)
C3A—C4A—H4AA	120.6	C13B—C12B—C11B	120.96 (15)
O3A—C5A—C4A	124.17 (15)	C14B—C13B—C12B	119.18 (17)
O3A—C5A—C6A	114.31 (15)	C14B—C13B—H13B	120.4
C4A—C5A—C6A	121.50 (16)	C12B—C13B—H13B	120.4
C1A—C6A—C5A	118.15 (14)	C13B—C14B—C15B	118.85 (17)
C1A—C6A—C9A	122.61 (13)	C13B—C14B—H14B	120.6
C5A—C6A—C9A	119.15 (14)	C15B—C14B—H14B	120.6
O2A—C7A—H7AA	109.5	C16B—C15B—C14B	118.07 (17)
O2A—C7A—H7AB	109.5	C16B—C15B—H15B	121.0
H7AA—C7A—H7AB	109.5	C14B—C15B—H15B	121.0
O2A—C7A—H7AC	109.5	N2B—C16B—C15B	124.15 (19)
H7AA—C7A—H7AC	109.5	N2B—C16B—H16B	117.9
H7AB—C7A—H7AC	109.5	C15B—C16B—H16B	117.9
O3A—C8A—H8AA	109.5	S1B—C17B—H17D	109.5
O3A—C8A—H8AB	109.5	S1B—C17B—H17E	109.5
H8AA—C8A—H8AB	109.5	H17D—C17B—H17E	109.5
O3A—C8A—H8AC	109.5	S1B—C17B—H17F	109.5
H8AA—C8A—H8AC	109.5	H17D—C17B—H17F	109.5
H8AB—C8A—H8AC	109.5	H17E—C17B—H17F	109.5
N1A—C9A—C6A	126.02 (14)	S1B—C18B—H18D	109.5
N1A—C9A—H9AA	117.0	S1B—C18B—H18E	109.5
C6A—C9A—H9AA	117.0	H18D—C18B—H18E	109.5
N1A—C10A—C11A	111.85 (13)	S1B—C18B—H18F	109.5
N1A—C10A—H10A	109.2	H18D—C18B—H18F	109.5
C11A—C10A—H10A	109.2	H18E—C18B—H18F	109.5
N1A—C10A—H10B	109.2	C1S—O1S—H1S	105 (2)
C11A—C10A—H10B	109.2	O1S—C1S—H1SD	109.5
H10A—C10A—H10B	107.9	O1S—C1S—H1SA	109.5
C12A—C11A—C10A	110.29 (14)	H1SD—C1S—H1SA	109.5
C12A—C11A—H11A	109.6	O1S—C1S—H1SB	109.5
C10A—C11A—H11A	109.6	H1SD—C1S—H1SB	109.5
C12A—C11A—H11B	109.6	H1SA—C1S—H1SB	109.5
C10A—C11A—H11B	109.6		
			
N1A—Mn1—O1A—C1A	30.38 (13)	N2A—C12A—C13A—C14A	0.1 (3)
N1A^i^—Mn1—O1A—C1A	−149.62 (13)	C11A—C12A—C13A—C14A	178.06 (19)
O4A—Mn1—O1A—C1A	−61.94 (13)	C12A—C13A—C14A—C15A	−0.4 (3)
O4A^i^—Mn1—O1A—C1A	118.05 (13)	C13A—C14A—C15A—C16A	0.4 (4)
C17A—S1A—O4A—Mn1	143.32 (9)	C12A—N2A—C16A—C15A	−0.2 (3)
C18A—S1A—O4A—Mn1	−113.81 (9)	C14A—C15A—C16A—N2A	−0.1 (4)
N1B^ii^—Mn2—O1B—C1B	−157.35 (15)	Mn2—O1B—C1B—C2B	161.78 (12)
N1B—Mn2—O1B—C1B	22.65 (15)	Mn2—O1B—C1B—C6B	−19.5 (2)
O4B^ii^—Mn2—O1B—C1B	−69.36 (15)	O1B—C1B—C2B—C3B	179.24 (15)
O4B—Mn2—O1B—C1B	110.64 (15)	C6B—C1B—C2B—C3B	0.5 (2)
C17B—S1B—O4B—Mn2	122.40 (9)	C7B—O2B—C3B—C2B	7.4 (3)
C18B—S1B—O4B—Mn2	−134.66 (11)	C7B—O2B—C3B—C4B	−173.44 (17)
Mn1—O1A—C1A—C2A	154.60 (11)	C1B—C2B—C3B—O2B	179.24 (16)
Mn1—O1A—C1A—C6A	−27.4 (2)	C1B—C2B—C3B—C4B	0.2 (3)
O1A—C1A—C2A—C3A	177.68 (15)	O2B—C3B—C4B—C5B	179.67 (16)
C6A—C1A—C2A—C3A	−0.4 (2)	C2B—C3B—C4B—C5B	−1.2 (3)
C7A—O2A—C3A—C2A	5.8 (3)	C8B—O3B—C5B—C4B	−2.3 (3)
C7A—O2A—C3A—C4A	−175.16 (17)	C8B—O3B—C5B—C6B	177.06 (16)
C1A—C2A—C3A—O2A	179.19 (16)	C3B—C4B—C5B—O3B	−179.19 (16)
C1A—C2A—C3A—C4A	0.2 (2)	C3B—C4B—C5B—C6B	1.5 (3)
O2A—C3A—C4A—C5A	−179.80 (16)	O1B—C1B—C6B—C5B	−178.92 (14)
C2A—C3A—C4A—C5A	−0.7 (3)	C2B—C1B—C6B—C5B	−0.2 (2)
C8A—O3A—C5A—C4A	2.2 (3)	O1B—C1B—C6B—C9B	−1.0 (2)
C8A—O3A—C5A—C6A	−179.41 (17)	C2B—C1B—C6B—C9B	177.68 (15)
C3A—C4A—C5A—O3A	179.69 (16)	O3B—C5B—C6B—C1B	179.83 (15)
C3A—C4A—C5A—C6A	1.4 (3)	C4B—C5B—C6B—C1B	−0.8 (2)
O1A—C1A—C6A—C5A	−176.92 (14)	O3B—C5B—C6B—C9B	1.8 (2)
C2A—C1A—C6A—C5A	1.1 (2)	C4B—C5B—C6B—C9B	−178.77 (15)
O1A—C1A—C6A—C9A	−0.6 (2)	C10B—N1B—C9B—C6B	−178.73 (15)
C2A—C1A—C6A—C9A	177.41 (14)	Mn2—N1B—C9B—C6B	0.0 (2)
O3A—C5A—C6A—C1A	179.97 (14)	C1B—C6B—C9B—N1B	10.1 (3)
C4A—C5A—C6A—C1A	−1.6 (2)	C5B—C6B—C9B—N1B	−172.01 (15)
O3A—C5A—C6A—C9A	3.5 (2)	C9B—N1B—C10B—C11B	−93.08 (17)
C4A—C5A—C6A—C9A	−178.08 (15)	Mn2—N1B—C10B—C11B	88.15 (14)
C10A—N1A—C9A—C6A	−178.90 (14)	N1B—C10B—C11B—C12B	−178.01 (13)
Mn1—N1A—C9A—C6A	−3.8 (2)	C16B—N2B—C12B—C13B	0.9 (3)
C1A—C6A—C9A—N1A	15.9 (2)	C16B—N2B—C12B—C11B	−178.7 (2)
C5A—C6A—C9A—N1A	−167.76 (15)	C10B—C11B—C12B—N2B	89.7 (2)
C9A—N1A—C10A—C11A	−93.76 (17)	C10B—C11B—C12B—C13B	−89.9 (2)
Mn1—N1A—C10A—C11A	90.96 (15)	N2B—C12B—C13B—C14B	−1.3 (3)
N1A—C10A—C11A—C12A	178.38 (14)	C11B—C12B—C13B—C14B	178.28 (17)
C16A—N2A—C12A—C13A	0.3 (3)	C12B—C13B—C14B—C15B	0.2 (3)
C16A—N2A—C12A—C11A	−177.81 (18)	C13B—C14B—C15B—C16B	1.1 (3)
C10A—C11A—C12A—N2A	88.9 (2)	C12B—N2B—C16B—C15B	0.6 (4)
C10A—C11A—C12A—C13A	−89.2 (2)	C14B—C15B—C16B—N2B	−1.6 (4)

Symmetry codes: (i) −*x*+1, −*y*+1, −*z*+1; (ii) −*x*+2, −*y*+1, −*z*.

### Hydrogen-bond geometry (Å, º)

**Table:**

*D*—H···*A*	*D*—H	H···*A*	*D*···*A*	*D*—H···*A*
O1*S*—H1*S*···O14	0.83 (2)	2.21 (4)	2.864 (4)	136 (5)
O1*S*—H1*S*···O13*A*	0.83 (2)	1.06 (3)	1.813 (7)	147 (6)
C7*A*—H7*AA*···O12^iii^	0.98	2.44	3.395 (3)	165
C7*A*—H7*AA*···O14*A*^iii^	0.98	2.49	3.415 (7)	157
C10*A*—H10*B*···O12^i^	0.99	2.57	3.408 (3)	142
C10*A*—H10*B*···O14*A*^i^	0.99	2.36	3.243 (8)	148
C17*A*—H17*B*···O12	0.98	2.69	3.173 (4)	112
C9*B*—H9*BA*···O11^iv^	0.95	2.60	3.405 (3)	143
C9*B*—H9*BA*···O11*A*^iv^	0.95	2.52	3.311 (7)	141
C16*A*—H16*A*···O12*A*^v^	0.95	2.66	3.563 (6)	160
C11*B*—H11*D*···O11*A*^iv^	0.99	2.57	3.380 (8)	139
C17*A*—H17*A*···O3*A*^vi^	0.98	2.55	3.455 (3)	154
C13*A*—H13*A*···O1*S*^i^	0.95	2.42	3.343 (4)	165
C18*A*—H18*B*···O4*A*^vi^	0.98	2.56	3.491 (3)	160
C17*B*—H17*F*···O4*B*^vii^	0.98	2.43	3.384 (2)	163
C7*B*—H7*BA*···O1*S*^viii^	0.98	2.51	3.445 (4)	160
C10*B*—H10*D*···O1*S*	0.99	2.57	3.428 (3)	146
C18*B*—H18*F*···O1*S*	0.98	2.62	3.402 (3)	137

Symmetry codes: (i) −*x*+1, −*y*+1, −*z*+1; (iii) *x*, *y*−1, *z*; (iv) *x*+1, *y*, *z*; (v) −*x*+1, −*y*+2, −*z*+1; (vi) −*x*, −*y*+1, −*z*+1; (vii) −*x*+1, −*y*+1, −*z*; (viii) −*x*+2, −*y*+2, −*z*.
